# Phase II Study Evaluating the Efficacy of Niraparib and Dostarlimab (TSR-042) in Patients with Recurrent/Metastatic Head and Neck Squamous Cell Carcinoma

**DOI:** 10.1158/2767-9764.CRC-25-0192

**Published:** 2025-06-09

**Authors:** Olga Zamulko, Vidhya Karivedu, Muhammad Kashif Riaz, Ilaina Monroe, Audrey Romano, Rachel Mulanda, Nicky Kurtzweil, Allie Forsythe, Casey L. Allen, Nusrat Harun, Jianmin Pan, Shesh Rai, Dalia El-Gamal, Trisha M. Wise-Draper

**Affiliations:** 1 Division of Hematology Oncology, Department of Internal Medicine, University of Cincinnati, Cincinnati, Ohio.; 2 Division of Hematology/Oncology, Duke University Medical Center, Durham, North Carolina.; 3 St. Elizabeth Healthcare, Edgewood, Kentucky.; 4 University of Cincinnati Cancer Center, Cincinnati, Ohio.; 5 Cytel, Cincinnati, Ohio.; 6 Biostatistics and Informatics Shared Resource, University of Cincinnati Cancer Center, Cincinnati, Ohio.; 7 Cancer Data Science Center, University of Cincinnati College of Medicine, Cincinnati, Ohio.; 8 Department of Biostatistics, Health Informatics and Data Sciences, University of Cincinnati College of Medicine, Cincinnati, Ohio.

## Abstract

**Purpose:**

Recurrent/metastatic head and neck squamous cell carcinoma (R/M HNSCC) portends a poor prognosis. DNA pathway repair mutations in HNSCC are associated with higher tumor mutational burden rates and immune checkpoint inhibitor response. PARP inhibitors (PARPi) induce ssDNA breaks and are efficacious in cancers with DNA repair defects. Thus, we designed a single-arm, open-label, phase II clinical trial to evaluate the combination of niraparib and dostarlimab in patients with R/M HNSCC.

**Patients and Methods:**

Patients with R/M HNSCC were treated with niraparib and dostarlimab until disease progression or unacceptable toxicity. The primary endpoint was the overall response rate and clinical benefit assessed by RECIST version 1.1. Using Simon’s two-step minimax design, 14 patients were planned to enroll in the first stage with a goal of overall clinical benefit of 50%.

**Results:**

Ten patients were enrolled. The majority were White males with a median age of 62.5. One patient had a PD-L1 combined positive score >20, a high tumor mutational burden, a *BRCA1* rearrangement, and an *ATRX* splice site mutation. Nine patients previously failed anti–PD-1/PD-L1 therapy. The best overall response rate was 10%, with a 20% clinical benefit (1 partial response, 1 stable disease). The trial was terminated early for futility as the goal clinical benefit could not be reached. At a median follow-up of 10.13 months, the median progression-free survival was 3.8 months, and the median overall survival was 10.1 months. The most common grade 3 or higher treatment-related adverse events were thrombocytopenia and hypertension.

**Conclusions:**

The combination of niraparib and dostarlimab did not achieve the primary endpoint of clinical benefit, but activity may be improved with biomarker-driven treatment and selected patients.

**Significance:**

Patients with R/M HNSCC that progress on PD-1 inhibitors have poor prognoses. PARPis cause ssDNA breaks that accumulate in cells with mutations in DNA damage repair pathways, leading to synthetic lethality. However, PARPi also inhibits glycogen synthase kinase-3β activity, leading to upregulated PD-L1, which is abrogated by PD-1 inhibitors. In this study, we combine niraparib (PARPi) with dostarlimab (anti–PD-L1) to evaluate clinical benefit in patients with R/M HNSCC.

## Introduction

According to the 2023 Surveillance, Epidemiology, and End Results Program, the incidence of head and neck cancer is 11.9 per 100,000 (1). For patients with early-stage disease (stage I or II), the survival rate is greater than 80% but rapidly declines to 30% to 50% for those diagnosed with late-stage disease (stage III or IV; ref. 2). Upon recurrence and/or metastasis, treatment is complicated by a lack of reliable salvage therapy. Targeted agents and immunotherapy have emerged as treatment modalities, with EGFR inhibitors and anti–PD-1 therapy being widely used as palliative treatment despite the relatively low response rates of 15% to 20%. Thus, the need exists for novel drug combinations with immunotherapy that are safe and effective.

PARP enzymes are DNA-binding enzymes that regulate single-strand break repair pathways (3). Unrepaired ssDNA breaks are converted to double-strand breaks, which are typically repaired by homologous recombination (HR); however, cells that are unable to repair DNA via HR rely on other error-prone pathways. PARP inhibitors (PARPi) have been studied in tumor cells with defects in HR as an opportunity to selectively kill those cells. For example, *BRCA* mutations are common in breast, ovarian, and prostate cancers. However, other non-BRCA mutant tumors share some of the same DNA repair defects, a phenomenon known as “BRCAness.” A landmark study published in 2006 revealed hypersensitivity to PARPi in cells with HR-related genes, DNA damage signaling genes, or Fanconi anemia–related genes (4). The prevalence of these mutations varies in head and neck squamous cell carcinoma (HNSCC) with more commonly mutated genes at rates of 17% (5).

The inherent link between DNA damage and immunity has been well studied. Mechanistically, PARPi causes ssDNA damage, which is sensed by pattern recognition receptors and leads to the activation of the cGAS-STING pathway, promoting the production of IFNs and other inflammatory cytokines that induce antitumor immunity (6). Previous preclinical studies showed that the combination of PARPi and anti–PD-1 therapy increased therapeutic efficacy over either therapy alone. Specifically, in breast cancer and animal models, PARP inhibition upregulates PD-L1 expression via inactivation of glycogen synthase kinase-3β, suggesting an intrinsic protective mechanism downstream of PARPi-induced killing (7). However, this effect was overcome by the introduction of anti–PD-L1 therapy in preclinical studies (8). Lastly, it has been shown that cells with high tumor mutational burden (TMB), including cells with DNA damage repair (DDR) mutations such as Fanconi Anemia (FA) and FA-related DNA repair pathways, have a better and longer duration of response to PD-1 inhibition (9). In light of this preclinical data, the synergistic effect of the combination of PARP and PD-1 inhibition has been evaluated in clinical trials in triple-negative breast cancer (TNBC), endometrial cancer, and non–small cell lung cancer (NSCLC; refs. 10–12), and clinical trials remain underway in cervical cancer (NCT04068753) and other cancer types. The overall response rate (ORR) was high (56%) with the combination in NSCLC (all PD-L1–positive) but lower at 21% and 14% in TNBC and endometrial cancer, respectively; none of the populations were selected based on the presence of a DDR mutation.

We hypothesized that the combination of PARPi and anti–PD-1 therapy would increase therapeutic efficacy over either therapy alone, thereby increasing response rates without overlapping toxicity in recurrent/metastatic HNSCC (R/M HNSCC). We designed a phase II clinical trial with the combination of niraparib, a highly selective PARP1 and PARP2 inhibitor, and dostarlimab (TSR-042), a humanized IgG4 monoclonal antibody that binds rapidly to PD-1 with high specificity and inhibits the binding of both PD-L1 and PD-L2. The primary endpoint was to determine the clinical benefit and overall best response in patients with R/M HNSCC receiving combination therapy with niraparib and dostarlimab. The trial was terminated early for futility as the expected response rate was not met despite some activity of the combination.

## Patients and Methods

### Study design and treatment

The study was an open-label, nonrandomized, single-arm phase II clinical trial of the combination of niraparib and dostarlimab in patients with R/M HNSCC who had failed at least one line of prior treatment and for whom there were no surgical or radiation curative options. The trial was registered on clinicaltrials.gov (NCT04313504). The trial complied with the Declaration of Helsinki, and good clinical practice was approved by the Institutional Review Board at the University of Cincinnati (IRB#2019-1161) and was monitored by the University of Cincinnati Cancer Center Data Safety Monitoring Board. All participants were required to sign a written informed consent.

Eligible patients were >18 years of age with histologically or cytologically confirmed R/M HNSCC who had failed at least one prior therapy, including surgery, radiotherapy, chemotherapy, and/or an anti–PD-1/PD-L1 inhibitor in the definitive or R/M setting; had an Eastern Cooperative Oncology Group performance status ≤2; had adequate organ function; and were able to swallow pills. Key exclusion criteria included nasopharyngeal and salivary gland tumors, autoimmune disease on immunosuppressive therapy, steroid use ≥10 mg daily, or any uncontrolled intercurrent illness. Importantly, patients must not have been exposed to both immunotherapy medication and PARPi in past treatments, but singular prior exposure was allowed.

All patients were evaluable for toxicity starting from the time of consent, and adverse events (AE) were graded according to the NCI Common Terminology for Adverse Events version 5.0. Only patients with measurable disease who had received at least one cycle of therapy and had their disease reevaluated were evaluable for response using RECIST version 1.1.

Niraparib, as a single agent given at 300 mg daily, was considered safe. However, when given in combination with pembrolizumab, in the phase I TOPACIO/Keynote-162 study (13), which enrolled patients with TNBC and recurrent ovarian cancer, the recommended phase II dose was ultimately determined to be 200 mg orally daily in combination with pembrolizumab. In several other early clinical trials, niraparib dosing of 200 mg daily was well tolerated when combined with checkpoint inhibition with established efficacy (10, 12). Therefore, given this historical data and an average body weight of 75 kg, a fixed daily dose of 200 mg oral niraparib was included in the current study.

Treatment began with niraparib 200 mg orally daily on day 1, followed by dostarlimab 500 mg intravenously every 3 weeks on day 8 of cycle 1 and day 1 of cycles 2 to 4. The dose of dostarlimab was changed to 1,000 mg intravenously every 6 weeks for cycles 5+ until discontinuation of treatment. See Supplementary Fig. S1 for the study schema.

### Biomarker analysis

Genomic mutational analysis for TMB, mismatch repair, and RNA expression analysis was performed as standard of care by submission to Caris Life Sciences on formalin-fixed, paraffin-embedded slides collected at screening. The following HR DDR pathologic gene mutations were evaluated by next-generation sequencing: *ARID1A*, *ATM*, *ATRX*, *BASP1*, *BATD1*, *BLM*, *BRCA1/2*, *BRIP1*, *CHEK1/2*, *FANCA/C/D2/E/F/G/L*, *MRE11A*, *NBM*, *PALB2*, *RAD50*, *RAD51*, *RAD51B*, and *WRN*. It was considered high if the value was ≥10 Mut/Mb for TMB. PD-L1 IHC was performed with the 22C3 antibody and reported as a combined positive score (CPS).

### Treatment response

The primary endpoint of the study was clinical benefit and ORR, which was measured using the best overall response by RECIST version 1.1. The response of target lesions was recorded as complete response (CR), partial response (PR), stable disease (SD), or progressive disease. The best overall response was defined as the best response from the start of treatment until disease progression and included patients with CR or PR, whereas clinical benefit included CR, PR, or SD. Note that patients were allowed to remain in the study at first progression, given that immunotherapy was used if the investigator felt the patient derived clinical benefit. Patients were reevaluated every 8 weeks for response. Imaging was performed during screening and prior to cycle 3, day 1, then 4 weeks after the initial documentation of response or progression to confirm assessment.

### Statistical analysis

Sample size was determined using Simon’s two-stage minimax design with a 0.05 significance threshold and 80% detection power. Under these assumptions, we planned to recruit a total of 23 patients. Fourteen patients were planned for the first stage. If at least seven patients developed a CR, PR, or SD (50% clinical benefit based on historical rates of single-agent PD-1/PD-L1 inhibitor monotherapy), as outlined above at the first 8-week scans, then the remaining nine patients would be enrolled; otherwise, the trial would be terminated. The secondary endpoints of the study included overall survival (OS) and progression-free survival (PFS), for which the Kaplan–Meier method was used to estimate median survival with a 95% confidence interval (CI). AEs, clinical benefit, and ORR were graded with tables and descriptive statistics.

### Data availability

The data generated in this study are included in the main article or Supplementary Data. Deidentified patient-level data are available upon request from the corresponding author.

## Results

Between December 2020 and November 2022, a total of 10 patients were enrolled. A consort diagram is available as Supplementary Fig. S2. The vast majority of those enrolled were male (90%) and White (90%). The median age was 62.5 years (range, 39–72). Primary disease sites included the oral cavity (20%) and oropharynx (80%). Eight (80%) patients received at least one prior line of treatment in the recurrent/metastatic setting, with all but one patient having previously failed a PD-1 or PD-L1 inhibitor (one patient received a PD-1 inhibitor in the curative intent setting). All demographic data are shown in Table 1. Enrolled patients were representative overall of the population (Supplementary Table S4).

**Table 1:** Patient demographics and disease characteristics

Patient characteristics	*n* = 10
Median age, years (range)	62.5 (39–72)
Sex, *N* (%)	
Male	9 (90)
Female	1 (10)
Race, *N* (%)	
White	9 (90)
African American	1 (10)
Ethnicity, *N* (%)	
Non-Hispanic	10 (100)
Smoking history, *N* (%)	
Smoker (>10 pack years)	5 (50)
Nonsmoker	5 (50)
Drinking history, *N* (%)	
Yes	5 (50)
No	5 (50)
ECOG, *N* (%)	
0	4 (40)
1	6 (60)
Primary disease site, *N* (%)	
Oral cavity	2 (20)
Oropharynx	8 (80)
Tumor classification, *N* (%)	
T1	3 (30)
T2	3 (30)
T2a	1 (10)
T4a	2 (20)
Tx	1 (10)
Lymph node classification, *N* (%)	
N1	5 (50)
N2	1 (10)
N2b	1 (10)
N3	1 (10)
N3b	2 (20)
Distant metastasis, *N* (%)	
M0	4 (40)
M1	6 (60)
P16 positive, *N* (%)	
Yes	5 (50)
No	5 (50)
PD-L1 positive, *N* (%)	
Yes	7 (70)
No	3 (30)
Prior lines of treatment^a^	
0	2 (20)
1	5 (50)
2	3 (30)
Types of prior treatment	
Curative intent	
Radiation	9 (90)
Platinum chemotherapy	5 (50)
PD-1/PD-L1 inhibitor	1 (10)
Anti–EGFR	3 (30)
Recurrent/metastatic	
Platinum chemotherapy	4 (40)
PD-1/PD-L1 inhibitor	8 (80)
Anti–EGFR	2 (20)

Note that Hispanic ethnicity and ECOG 2 were not included in the table as zero patients matched those demographics.

Abbreviation: ECOG, Eastern Cooperative Oncology Group performance status.

a In the recurrent/metastatic setting, excluding palliative radiation; one patient received both pembrolizumab and then nivolumab monotherapy, which was counted as one line of therapy.

Of the 10 patients enrolled in the trial, 7 patients were evaluable for response by imaging, and 3 additional patients were deemed clinically progressed although they did not have scan confirmation. Of the 10 evaluable patients, there were 0 CR, 1 PR, 1 SD, and 8 progressive disease, making the ORR 10%, and the clinical benefit was 20%. The patient with PR was PD-L1 positive (CPS 10), had low TMB, and had no DDR gene alterations. The patient with SD was PD-L1 positive with CPS 10. Molecular profiling was unavailable. Two patients did have an identifiable DDR deficiency, which included a *BRCA1* rearrangement and an *ATRX* splice site mutation; both patients progressed on treatment. Table 2 represents the breakdown of available biomarkers. Note that molecular data were unavailable for two patients due to insufficient tissue or indeterminate results for sequencing. One patient had negative PD-L1 by SP124 antibody and thus was not included in CPS reporting. In general, there was no correlation between CPS and the presence or absence of DDR mutations in the limited sample population. Given that there were only two enrolled patients who derived clinical benefit, the trial was terminated early for futility as it was not possible to reach 7 out of 14 responses, including SD, or a clinical benefit of 50%.

**Table 2:** Molecular biomarkers

Biomarker	*n* (%)
TMB	Total *n* = 8
Low (0–9 mut/Mb)	7 (87.5)
High (≥10 mut/Mb)	1 (12.5)
PD-L1 CPS	Total *n* = 9
<1	0 (0)
≥1–20	8 (89)
≥20	1 (11)
DDR	Total *n* = 8
Yes	2 (25)
No	6 (75)

**Figure 1:**
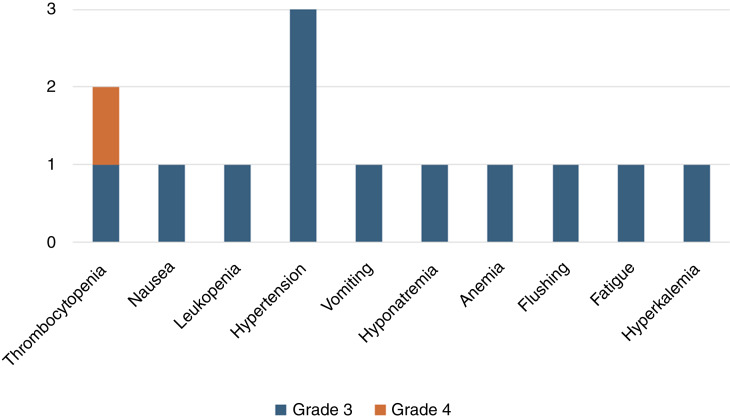
Grade 3 or higher treatment-related AEs. Thrombocytopenia and hypertension were the most common. One grade 4 event of thrombocytopenia occurred. There were no grade 5 treatment-related AEs.

Of the 12 consented patients, including the two screen failures, 11 patients experienced at least 1 AE. Nausea, weight loss, and fatigue were the most common grade 1 to 2 AEs. Grade 3 or higher treatment-related AEs occurred in 70% of patients (7/10), of which thrombocytopenia and hypertension were the most common, as shown in Fig. 1, which were attributed to niraparib. One grade 4 niraparib-related AE occurred (thrombocytopenia). A full list of niraparib-related AEs is available in Supplementary Table S2. Dostarlimab immune–related AEs included grade 3 to 4 nausea, fatigue, hyperkalemia, hyponatremia, adrenal insufficiency, and flushing (Supplementary Table S3). Three patients required a dose reduction in niraparib due to thrombocytopenia (2) and acute renal failure (1). An additional two patients required a niraparib dose hold due to thrombocytopenia and anemia, but neither patient restarted the medication due to declining disease status. A complete list of AEs is available in Supplementary Table S1, and AEs are grouped by categories in Supplementary Fig. S3, whereas all treatment-related AEs are represented in Supplementary Fig. S4.

**Figure 2:**
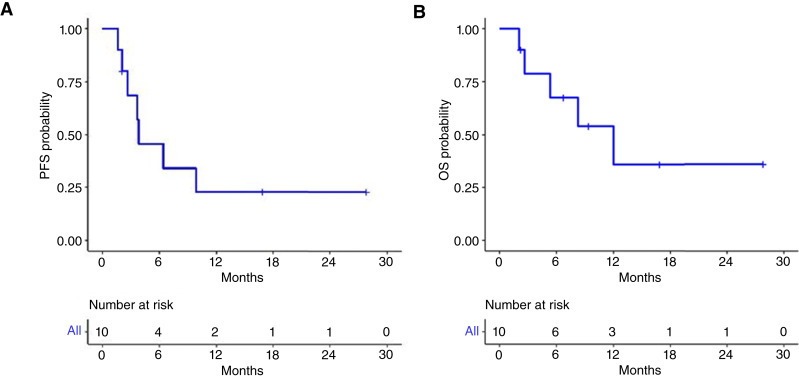
Kaplan–Meier survival curves. **A,** Median PFS was 3.8 months. **B,** Median OS was 10.1 months.

The secondary endpoints of PFS and OS are shown in Fig. 2. At a median follow-up of 10.13 months, the median PFS was 3.8 months (95% CI, 1.63–NA). The median OS was 10.1 months (95% CI, 2.06–NA; ref. 14). The upper limit CIs are not available due to limited events to obtain reliable data.

## Discussion

Treatment of R/M HNSCC continues to be a challenge; thus, the need for novel combination therapy exists. This single-arm, open-label phase II clinical trial was designed to evaluate the combination of dostarlimab, an anti–PD-1 medication, with niraparib, a PARPi, in R/M HNSCC. A total of 10 out of 23 planned patients were enrolled in the trial. The low accrual rate was, in part, due to severe dysphagia in many patients with R/M HNSCC being unable to swallow the niraparib pills. The trial was terminated for futility, as mathematically, 7 out of 14 responses, including SD (clinical benefit of 50%), were not achievable given that only 2 patients derived clinical benefit in the first 10 enrolled.

Notably, all but one patient enrolled in the trial were previously exposed to immunotherapy. Given that response rates for immunotherapy monotherapy after failure of curative intent treatment or after first-line R/M nonimmunotherapy-containing regimens (extreme) range from 15% to 25% and that immunotherapy regimens after failure of previous immunotherapy range closer to 10% to 15%, the 20% clinical benefit derived from this current combination may still be favorable and may be worth pursuing in selected patients (15, 16). Furthermore, only one patient had a high PD-L1 CPS >20. Therefore, a redesign of the study with PD-1 inhibitor–naïve R/M HNSCC with high PD-L1 CPS may lead to higher response rates. Further, the patients were not selected for DDR deficiencies. Although a *BRCA1* rearrangement and an *ATRX* splice site mutation were found in two distinct patients, the sample size was not sufficient to test this hypothesis. Given that DDR deficiencies increase PARPi efficacy, a biomarker-selected trial may result in enhanced activity.

Overall, median OS was near that of previous studies with pembrolizumab alone in PD-1 inhibitor–naïve patients, at 11.5 months. Median PFS, however, was shorter, at approximately 4 months, compared with 9 months in historical controls (17). The small sample size and heavily pretreated population, especially with anti–PD-1, may once again be responsible for these results.

Studies have reported the rate of grade 3 to 4 AEs of 11% and 37% in PD-1 inhibitors and PARPi, respectively. The combination of PARPi and immunotherapy resulted in grade 3 or higher AEs in 70% of patients, which was above historical data and the 60% threshold per protocol although higher rates have been seen in NSCLC (10). This may be explained, in part, by the fact that this population was heavily pretreated. Any further examination of this particular combination would need careful evaluation of further safety concerns in this population.

Despite disappointing results in our heavily pretreated population, PARPi shows promise in treatment–naïve patients with HNSCC in combination with immunotherapy and chemotherapy. A clinical trial studying olaparib in combination with pembrolizumab and carboplatin in the first-line treatment of R/M HNSCC showed an ORR of 50% (18).

Patient accrual was a significant limitation of this study, specifically challenges with swallowing medication in this population, which led to a small sample size limiting analysis. Furthermore, patients were heavily pretreated, with the vast majority of participants previously receiving immunotherapy and not selected based on PD-L1 expression or DDR deficiencies. Regardless, the synergistic effect of PARP inhibition with anti–PD-1 therapy has shown activity in other cancer types, and emerging results are positive in R/M HNSCC when used in the frontline setting. Further evaluation of this promising combination treatment is thus warranted to improve outcomes in a historically unresponsive disease.

## Supplementary Material

Fig S1 Study design schema

Fig S2 Consort flow diagram of participants through each stage of the clinical trial

Fig S3 Bar graph of all adverse events

Fig S4 Bar graph of treatment related adverse events

Supplementary Data Fig Info Supplemental Figure Titles

Supplementary Table S1 All adverse events on trial.

Supplementary Table S2 Niraparib treatment-related events on trial.

Supplementary Table S3 Dostarlimab treatment-related adverse events on trial.

Supplementary Table S4 Representativeness of study participants
