# Supplementary figures and images for: Phase II Study Evaluating the Efficacy of Niraparib and Dostarlimab (TSR-042) in Patients with Recurrent/Metastatic Head and Neck Squamous Cell Carcinoma
Source: Cancer Res Commun. 2025 Jun 9;5(6):939–44. doi: 10.1158/2767-9764.CRC-25-0192 (PMC12146980; doi:10.1158/2767-9764.CRC-25-0192)

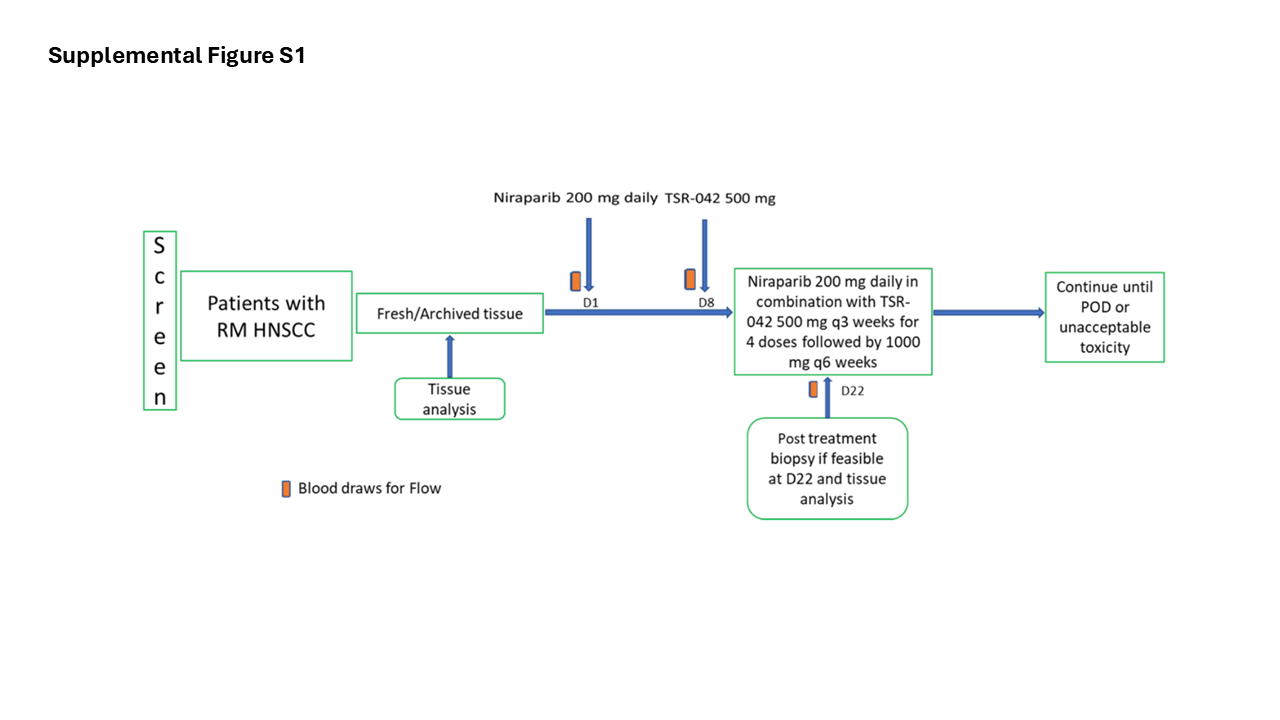

Supplement: Fig S1 — Study design schema [file crc-25-0192_fig_s1_suppsf1.png]

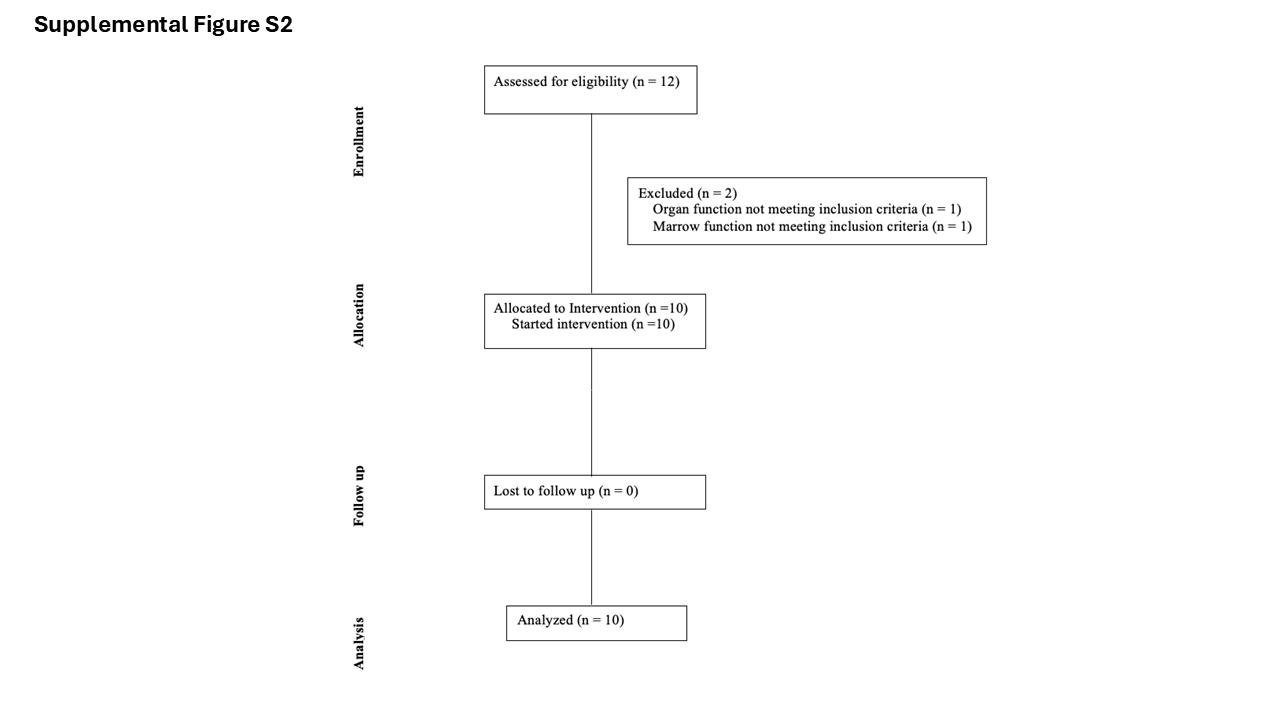

Supplement: Fig S2 — Consort flow diagram of participants through each stage of the clinical trial [file crc-25-0192_fig_s2_suppsf2.png]

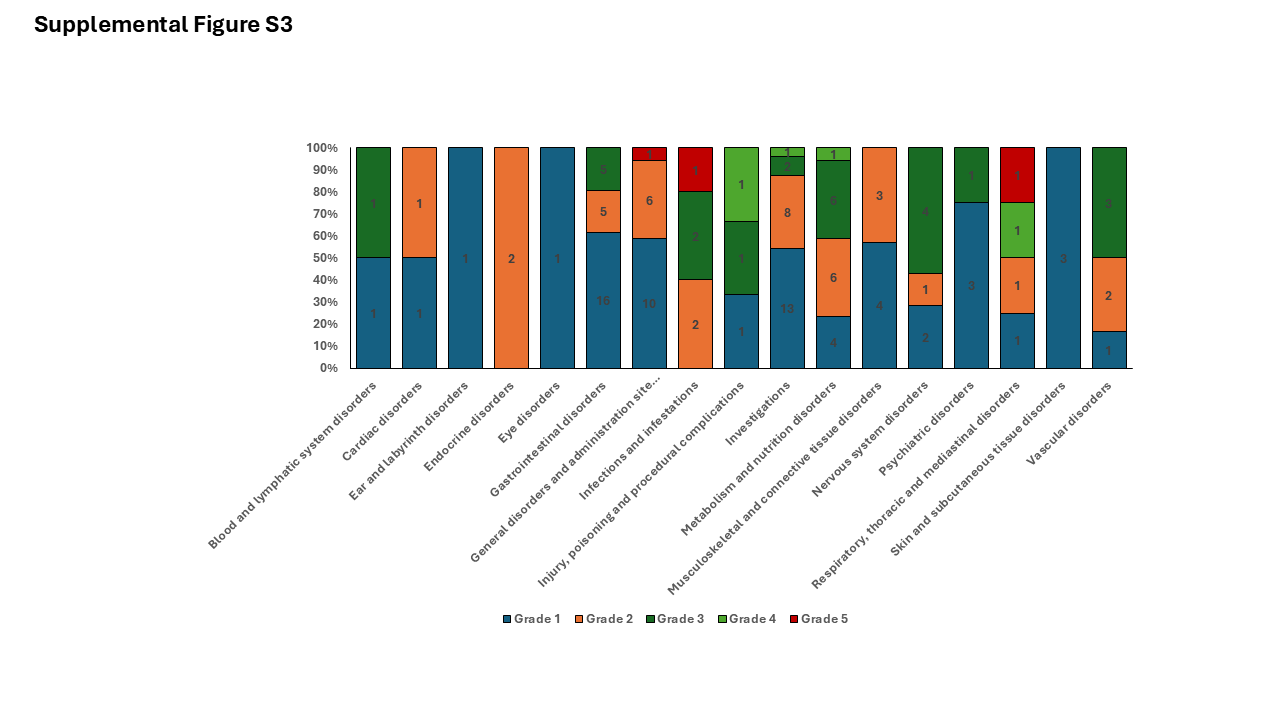

Supplement: Fig S3 — Bar graph of all adverse events [file crc-25-0192_fig_s3_suppsf3.png]

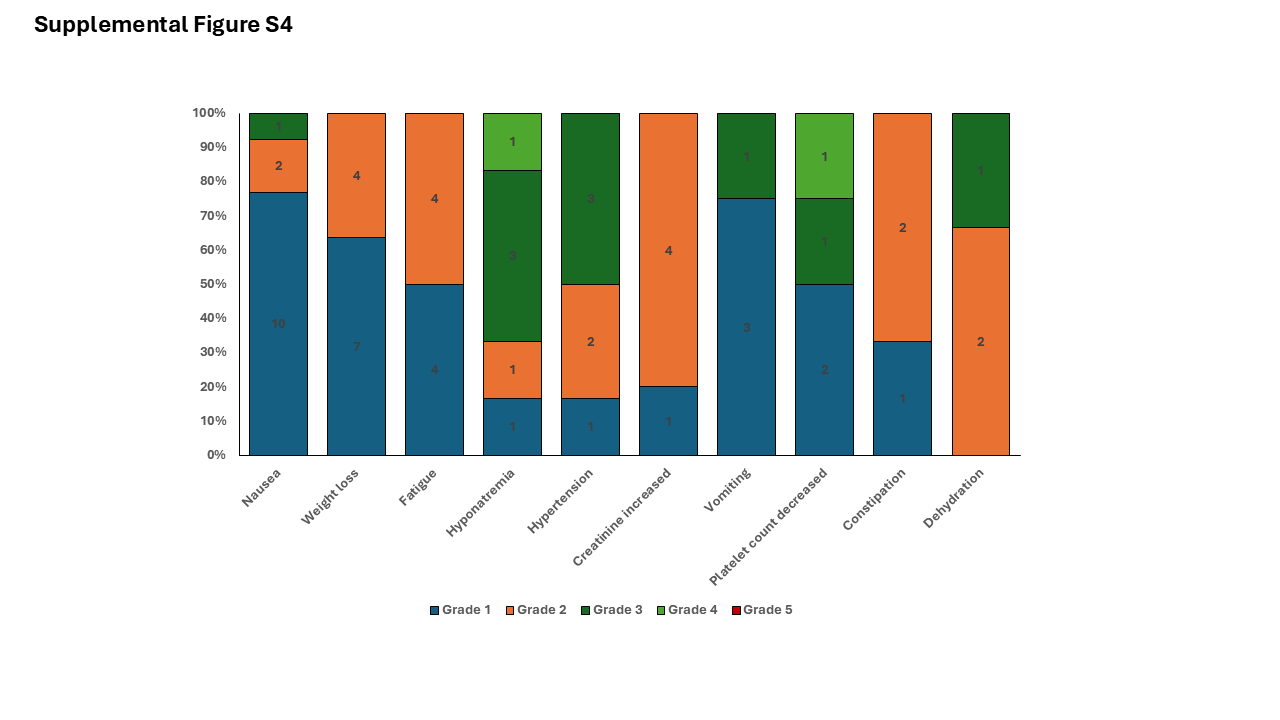

Supplement: Fig S4 — Bar graph of treatment related adverse events [file crc-25-0192_fig_s4_suppsf4.png]
