# Supplementary material for: Phase II Study Evaluating the Efficacy of Niraparib and Dostarlimab (TSR-042) in Patients with Recurrent/Metastatic Head and Neck Squamous Cell Carcinoma
Source: Cancer Res Commun. 2025 Jun 9;5(6):939–44. doi: 10.1158/2767-9764.CRC-25-0192 (PMC12146980; doi:10.1158/2767-9764.CRC-25-0192)
Supplement: Supplementary Data Fig Info — Supplemental Figure Titles [file crc-25-0192_supplementary_data_fig_info_suppsdf.docx]

**Supplemental Figure S1:** Study design schema.

**Supplemental Figure S2**: Consort flow diagram of participants through each stage of the clinical trial.

**Supplemental Figure S3**: Bar graph of all adverse events.

**Supplemental Figure S4:** Bar graph of treatment related adverse events.
