# Supplementary material for: Phase II Study Evaluating the Efficacy of Niraparib and Dostarlimab (TSR-042) in Patients with Recurrent/Metastatic Head and Neck Squamous Cell Carcinoma
Source: Cancer Res Commun. 2025 Jun 9;5(6):939–44. doi: 10.1158/2767-9764.CRC-25-0192 (PMC12146980; doi:10.1158/2767-9764.CRC-25-0192)
Supplement: Supplementary Table S1 — All adverse events on trial. [file crc-25-0192_supplementary_table_s1_suppst1.docx]

**Supplemental Table S1:** All adverse events on trial.

| **CTCAE Category; Term** | **Grade 1 - 2** | **Grade 3 - 4** | **Grade 5** |
| --- | --- | --- | --- |
| **Blood and lymphatic system disorders** |  |  |  |
| Anemia | 0 (00) | 1 (10) | 0 (00) |
| **Cardiac disorders** |  |  |  |
| Palpitations | 1 (10) | 0 (00) | 0 (00) |
| Sinus tachycardia | 2 (20) | 0 (00) | 0 (00) |
| **Ear and labyrinth disorders** |  |  |  |
| Ear pain | 1 (10) | 0 (00) | 0 (00) |
| **Endocrine disorders** |  |  |  |
| Adrenal insufficiency | 0 (00) | 1 (10) | 0 (00) |
| Hypothyroidism | 2 (20) | 0 (00) | 0 (00) |
| **Eye disorders** |  |  |  |
| Blurred vision | 2 (20) | 0 (00) | 0 (00) |
| **Gastrointestinal disorders** |  |  |  |
| Abdominal pain | 1 (10) | 0 (00) | 0 (00) |
| Bloating | 1 (10) | 0 (00) | 0 (00) |
| Constipation | 4 (40) | 0 (00) | 0 (00) |
| Diarrhea | 1 (10) | 0 (00) | 0 (00) |
| Dysphagia | 1 (10) | 2 (20) | 0 (00) |
| Nausea | 7 (70) | 1 (10) | 0 (00) |
| Oral hemorrhage | 0 (00) | 2 (20) | 0 (00) |
| Oral pain | 1 (10) | 0 (00) | 0 (00) |
| Sore throat | 1 (10) | 0 (00) | 0 (00) |
| Toothache | 1 (10) | 0 (00) | 0 (00) |
| Vomiting | 4 (40) | 1 (10) | 0 (00) |
| Gastrointestinal disorders –Other (coughing up gastric  content) | 1 (10) | 0 (00) | 0 (00) |
| **General disorders and administration site conditions** |  |  |  |
| Cervical adenopathy | 2 (20) | 0 (00) | 0 (00) |
| Chills | 2 (20) | 0 (00) | 0 (00) |
| Edema face | 1 (10) | 0 (00) | 0 (00) |
| Fatigue | 6 (60) | 1 (10) | 0 (00) |
| Flu like symptoms | 2 (20) | 0 (00) | 0 (00) |
| Lightheadedness | 1 (10) | 0 (00) | 0 (00) |
| Neck edema | 1 (10) | 0 (00) | 0 (00) |
| Pain | 2 (20) | 0 (00) | 0 (00) |
| **Infections and infestations** |  |  |  |
| Jaw infection | 1 (10) | 0 (00) | 0 (00) |
| Lung infection | 0 (00) | 1 (10) | 1 (10) |
| Thrush | 2 (20) | 0 (00) | 0 (00) |
| Wound infection | 1 (10) | 1 (10) | 0 (00) |
| **Injury, poisoning and procedural complications** |  |  |  |
| Bruising | 1 (10) | 0 (00) | 0 (00) |
| Tracheal obstruction | 0 (00) | 1 (10) | 0 (00) |
| Tracheostomy site bleeding | 0 (00) | 1 (10) | 0 (00) |
| **Investigations** |  |  |  |
| Alkaline phosphatase increased | 2 (20) | 0 (00) | 0 (00) |
| Aspartate aminotransferase increased | 1 (10) | 0 (00) | 0 (00) |
| BUN Increased | 3 (30) | 0 (00) | 0 (00) |
| Creatinine increased | 3 (30) | 0 (00) | 0 (00) |
| Platelet count decreased | 2 (20) | 1 (10) | 0 (00) |
| Thyroid stimulating hormone increased | 1 (10) | 0 (00) | 0 (00) |
| Weight loss | 6 (60) | 0 (00) | 0 (00) |
| White blood cell decreased | 0 (00) | 1 (10) | 0 (00) |
| **Metabolism and nutritional disorders** |  |  |  |
| Anorexia | 2 (20) | 1 (10) | 0 (00) |
| Appetite change | 1 (10) | 0 (00) | 0 (00) |
| Dehydration | 3 (30) | 1 (10) | 0 (00) |
| Hyperglycemia | 1 (10) | 0 (00) | 0 (00) |
| Hyperkalemia | 0 (00) | 1 (10) | 0 (00) |
| Hypokalemia | 1 (10) | 0 (00) | 0 (00) |
| Hypomagnesemia | 1 (10) | 0 (00) | 0 (00) |
| Hyponatremia | 1 (10) | 2 (20) | 0 (00) |
| **Musculoskeletal and connective tissue disorders** |  |  |  |
| Arthralgia | 1 (10) | 0 (00) | 0 (00) |
| Back pain | 1 (10) | 0 (00) | 0 (00) |
| Jaw cramps | 1 (10) | 0 (00) | 0 (00) |
| Muscle weakness lower limb | 1 (10) | 0 (00) | 0 (00) |
| Neck stiffness | 2 (20) | 0 (00) | 0 (00) |
| Trismus | 0 (00) | 1 (10) | 0 (00) |
| Musculoskeletal and connective tissue disorder- Other  (difficulty chewing) | 1 (10) | 0 (00) | 0 (00) |
| **Nervous system disorders** |  |  |  |
| Cognitive disturbance | 0 (00) | 1 (10) | 0 (00) |
| Headache | 2 (20) | 0 (00) | 0 (00) |
| Memory impairment | 1 (10) | 0 (00) | 0 (00) |
| **Psychiatric disorders** |  |  |  |
| Agitation | 1 (10) | 0 (00) | 0 (00) |
| Anxiety | 2 (20) | 0 (00) | 0 (00) |
| Depression | 0 (00) | 1 (10) | 0 (00) |
| Hallucinations | 1 (10) | 0 (00) | 0 (00) |
| Insomnia | 1 (10) | 0 (00) | 0 (00) |
| **Renal and urinary disorders** |  |  |  |
| Chronic kidney disease | 1 (10) | 0 (00) | 0 (00) |
| Difficulty urinating | 1 (10) | 0 (00) | 0 (00) |
| **Respiratory, thoracic and mediastinal disorders** |  |  |  |
| Aspiration | 0 (00) | 0 (00) | 1 (10) |
| Aspiration pneumonia | 0 (00) | 1 (10) | 0 (00) |
| Bronchial stricture | 1 (10) | 0 (00) | 0 (00) |
| Cough | 2 (20) | 0 (00) | 0 (00) |
| Dyspnea | 4 (40) | 0 (00) | 0 (00) |
| Hoarseness | 1 (10) | 0 (00) | 0 (00) |
| Sinusitis | 1 (10) | 0 (00) | 0 (00) |
| Sore throat | 1 (10) | 0 (00) | 0 (00) |
| Tachypnea | 1 (10) | 0 (00) | 0 (00) |
| **Skin and subcutaneous tissue disorders** |  |  |  |
| Alopecia | 2 (20) | 0 (00) | 0 (00) |
| Dry skin | 1 (10) | 0 (00) | 0 (00) |
| Hyperhidrosis | 1 (10) | 0 (00) | 0 (00) |
| Pedunculated lesion | 1 (10) | 0 (00) | 0 (00) |
| Rash acneiform | 1 (10) | 0 (00) | 0 (00) |
| **Surgical and medical procedures** |  |  |  |
| Teeth extractions | 1 (10) | 0 (00) | 0 (00) |
| **Vascular disorders** |  |  |  |
| Flushing | 0 (00) | 1 (10) | 0 (00) |
| Hypertension | 0 (00) | 3 (30) | 0 (00) |
| Hypotension | 0 (00) | 1 (10) | 0 (00) |
