# Supplementary material for: Phase II Study Evaluating the Efficacy of Niraparib and Dostarlimab (TSR-042) in Patients with Recurrent/Metastatic Head and Neck Squamous Cell Carcinoma
Source: Cancer Res Commun. 2025 Jun 9;5(6):939–44. doi: 10.1158/2767-9764.CRC-25-0192 (PMC12146980; doi:10.1158/2767-9764.CRC-25-0192)
Supplement: Supplementary Table S2 — Niraparib treatment-related events on trial. [file crc-25-0192_supplementary_table_s2_suppst2.docx]

**Supplemental Table S2:** Niraparib treatment-related events on trial.

| **CTCAE Category; Term** | **Grade 1 - 2** | **Grade 3 - 4** |
| --- | --- | --- |
| **Blood and lymphatic system disorders** |  |  |
| Anemia | 1 (10) | 1 (10) |
| **Cardiac disorders** |  |  |
| Palpitations | 1 (10) | 0 (00) |
| **Gastrointestinal disorders** |  |  |
| Nausea | 6 (60) | 1 (10) |
| Oral pain | 1 (10) | 0 (00) |
| Sore throat | 1 (10) | 0 (00) |
| Vomiting | 2 (20) | 1 (10) |
| **General disorders and administration site conditions** |  |  |
| Chills | 1 (10) | 0 (00) |
| Fatigue | 6 (60) | 1 (10) |
| **Injury, poisoning and procedural complications** |  |  |
| Bruising | 1 (10) | 0 (00) |
| **Investigations** |  |  |
| Alkaline phosphatase increased | 1 (10) | 0 (00) |
| Aspartate aminotransferase increased | 1 (10) | 0 (00) |
| Platelet count decreased | 2 (20) | 1 (10) |
| Weight loss | 1 (10) | 0 (00) |
| White blood cell decreased | 0 (00) | 1 (10) |
| **Metabolism and nutritional disorders** |  |  |
| Anorexia | 3 (30) | 0 (00) |
| Appetite change | 1 (10) | 0 (00) |
| Dehydration | 1 (10) | 0 (00) |
| Hyponatremia | 1 (10) | 0 (00) |
| **Nervous system disorders** |  |  |
| Memory impairment | 1 (10) | 0 (00) |
| **Psychiatric disorders** |  |  |
| Agitation | 1 (10) | 0 (00) |
| Anxiety | 2 (20) | 0 (00) |
| Insomnia | 1 (10) | 0 (00) |
| **Respiratory, thoracic and mediastinal disorders** |  |  |
| Dyspnea | 1 (10) | 0 (00) |
| **Skin and subcutaneous tissue disorders** |  |  |
| Alopecia | 1 (10) | 0 (00) |
| Dry skin | 1 (10) | 0 (00) |
| **Vascular disorders** |  |  |
| Hypertension | 0 (00) | 2 (20) |
