# Supplementary material for: Phase II Study Evaluating the Efficacy of Niraparib and Dostarlimab (TSR-042) in Patients with Recurrent/Metastatic Head and Neck Squamous Cell Carcinoma
Source: Cancer Res Commun. 2025 Jun 9;5(6):939–44. doi: 10.1158/2767-9764.CRC-25-0192 (PMC12146980; doi:10.1158/2767-9764.CRC-25-0192)
Supplement: Supplementary Table S3 — Dostarlimab treatment-related adverse events on trial. [file crc-25-0192_supplementary_table_s3_suppst3.docx]

**Supplemental Table S3:** Dostarlimab treatment-related adverse events on trial.

| **CTCAE Category; Term** | **Grade 1 - 2** | **Grade 3 - 4** |
| --- | --- | --- |
| **Endocrine disorders** |  |  |
| Adrenal insufficiency | 0 (00) | 1 (10) |
| Hyperthyroidism | 1 (10) | 0 (00) |
| Hypothyroidism | 2 (20) | 0 (00) |
| **Gastrointestinal disorders** |  |  |
| Nausea | 2 (20) | 1 (10) |
| Oral pain | 1 (10) | 0 (00) |
| **General disorders and administration site conditions** |  |  |
| Fatigue | 5 (50) | 1 (10) |
| Flu like symptoms | 1 (10) | 0 (00) |
| **Investigations** |  |  |
| Aspartate aminotransferase increased | 1 (10) | 0 (00) |
| **Metabolism and nutrition disorders** |  |  |
| Anorexia | 1 (10) | 0 (00) |
| Hyperkalemia | 0 (00) | 1 (10) |
| Hyponatremia | 0 (00) | 1 (10) |
| **Musculoskeletal and connective tissue disorders** |  |  |
| Arthralgia | 1 (10) | 0 (00) |
| Neck stiffness | 1 (10) | 0 (00) |
| Musculoskeletal and connective tissue disorders – Other  (difficulty chewing) | 1 (10) | 0 (00) |
| **Nervous system disorders** |  |  |
| Memory impairment | 1 (10) | 0 (00) |
| **Psychiatric disorders** |  |  |
| Agitation | 1 (10) | 0 (00) |
| **Skin and subcutaneous tissue disorders** |  |  |
| Dry skin | 1 (10) | 0 (00) |
| Hyperhidrosis | 1 (10) | 0 (00) |
| **Vascular disorders** |  |  |
| Flushing | 0 (00) | 1 (10) |
