# Supplementary material for: Phase II Study Evaluating the Efficacy of Niraparib and Dostarlimab (TSR-042) in Patients with Recurrent/Metastatic Head and Neck Squamous Cell Carcinoma
Source: Cancer Res Commun. 2025 Jun 9;5(6):939–44. doi: 10.1158/2767-9764.CRC-25-0192 (PMC12146980; doi:10.1158/2767-9764.CRC-25-0192)
Supplement: Supplementary Table S4 — Representativeness of study participants [file crc-25-0192_supplementary_table_s4_suppst4.docx]

**Supplemental Table S4: Representativeness of study participants.**

| Cancer type/subtype/stage/condition | Head and neck squamous cell carcinoma |
| --- | --- |
| Considerations related to: |  |
| Sex | Globally, HNSCC is more common in males than females in approximately a 3:1 ratio. Our study also shows a male predominance, however, with only one female enrolled in the trial, conclusions based on sex exclusively should be avoided. |
| Age | According to 2022 SEER data, the median age at diagnosis of HNSCC is 64 years old. This is comparable to our study population with a median age of 62.5. |
| Race/ethnicity | HNSCC is most common in non-Hispanic patients. All 10 patients enrolled were non-Hispanic. It is also most common in White patients, which is represented in our study as well. HNSCC occurs in 5/100,000 (0.00005%) Black patients. We enrolled 1 patient (10%) with both demographics. |
| Geography | HNSCC is the 7^th^ most common cancer globally. Incidence rates are highest in India, followed by South and Southeast Asia, Central and Eastern Europe, and South America. In the USA, HNSCC accounts for approximately 3% of all malignancies. |
| Other considerations | Key exclusion criteria in this trial included nasopharyngeal carcinoma, thus these patients are not represented in the results. |
| Overall representativeness of this study | Overall, the study is representative of the general HNSCC population, which is most commonly non-Hispanic, White males greater than 50 years of age. |
